# Maternal practices and awareness of diarrhea management in children under-five: evidence from Pune, India

**DOI:** 10.3389/fpubh.2025.1673617

**Published:** 2025-12-08

**Authors:** Sandhya A. Shukla, Sonali P. Suryawanshi, Priti P. Dhande

**Affiliations:** Department of Pharmacology, Bharati Vidyapeeth (Deemed to be University) Medical College, Pune, India

**Keywords:** diarrhea, children, India, knowledge, practice, ORS, zinc

## Abstract

**Introduction:**

Diarrhea remains a leading cause of morbidity and mortality among children under-five in developing countries. Mothers’ knowledge and practices play a critical role in early home management and prevention.

**Objectives:**

To assess the knowledge, attitudes, and practices (KAP) of mothers of under-five children regarding the prevention and management of diarrhea in Pune, India.

**Methods:**

A cross-sectional, questionnaire-based survey was conducted among 122 mothers attending the pediatric outpatient department of a tertiary care hospital. Data on sociodemographic characteristics, knowledge, attitudes, and practices related to diarrhea were collected and analyzed using descriptive statistics and Chi-square tests.

**Results:**

Most mothers (67.2%) correctly defined diarrhea, and nearly all (99.18%) identified contaminated water as a major cause. Awareness of ORS was high, with 95.9% knowing its correct preparation. However, only 28.7% recognized the role of zinc supplementation. Higher education, employment, and income were significantly associated with better knowledge (*p* < 0.05), but not with practice scores. A weak positive correlation (*r* = 0.34) was found between knowledge and practice. About 27% followed all three WHO-recommended home-based treatments (ORS, increased fluids, continued feeding), while 3% relied solely on antibiotics.

**Conclusion:**

Mothers demonstrated good awareness of diarrhea causes and ORS use but lacked adequate knowledge of zinc supplementation. Health education efforts should target these gaps to promote complete and evidence-based home management practices.

## Introduction

Worldwide, diarrhea remains a leading cause of mortality after acute respiratory infections among children below 5 years and is also a significant contributor to malnutrition (1–3). According to the World Health Organization (WHO), three or more watery stools in a day, or any stool that occurs more frequently than usual for a child or mothers see as odd, is considered diarrhea. Diarrhea in children has become a major concern in India. Despite various schemes and plans to control it, mortality remains alarming among children below 5 years in India (4).

In developing countries, children below five typically suffer around three episodes of diarrhea a year (1, 5). Diarrhea risk is high in underdeveloped countries (6). Main causes include inadequate water, unhygienic practices, poor breastfeeding, and nutritional deficiencies, especially zinc and vitamin A (7). Proper management at health facilities and at home is key to reducing severity and deaths (8). United Nations Children’s Fund (UNICEF), WHO, and the Integrated Management of Childhood Illness have promoted home diarrhea management.

Diarrhea is preventable by hygienic practices, safe water, early recognition of dehydration, and rapid rehydration with oral rehydration solution (ORS) and home-available fluids. For children under five, healthy eating and good hygiene can reduce diarrhea-related morbidity and mortality (9, 10). The WHO acronym ‘WASH’ (water, sanitation, hygiene), rotavirus vaccination, exclusive breastfeeding, vitamin A supplementation, and proper handwashing are key preventive strategies (11).

Children in developing countries face higher mortality due to lack of timely treatment with zinc and ORS (7). Lack of health education is a major barrier (12). Mothers, as primary caregivers, play a crucial role, but their lack of knowledge, poor practices, and misconceptions often result in severe dehydration (13, 14).

Given mothers’ central role in diarrhea management, a joint WHO-UNICEF statement highlights the need to understand their current knowledge, attitude, and practices (15).

Hence current study was conducted to assess mothers’ Knowledge, Attitude, and Practice (KAP) in relation to managing and preventing diarrhea in children under five.

## Materials and methods

A descriptive cross-sectional study was conducted in the Pediatric Outpatient Department of a tertiary care teaching hospital in Pune, India, from May 2023 to December 2023 after the institutional ethics committee approval (REF: BVDUMC/IEC/133). The study focused on mothers of children under 5 years, as a primary caregiver and they play a crucial role in diarrhea prevention and management.

### Study population and sample size

Mothers aged 18–40 years, with a child experiencing diarrhea during enrollment or within the 2 weeks prior, and willing to participate, were included. The sample size was calculated using the single population proportion formula based on a 76% prevalence of maternal knowledge about diarrhea management reported in a previous Indian study (16). Assuming a 10% margin of error (*d* = 7.6), a sample size of 121.31 was obtained and rounded to 122 mothers.

### Data collection procedure and tool

Following institutional ethics committee approval, written informed consent was obtained. Data were collected using a structured, pretested questionnaire adapted from previous literature (4, 14). The tool was validated by a senior pediatrician and piloted among 15 mothers for clarity.

The questionnaire (Supplementary Table 1) comprised four sections: (A) socio-demographic variables (age, education, parity, occupation, family income); (B) The knowledge assessment component consisted of seven multiple-choice items evaluating respondents’ understanding of the concept of childhood diarrhea, its causative factors, clinical manifestations, transmission routes, potential complications, and preventive measures. Each correct answer was allotted with a score of 1, while incorrect answers received 0. The cumulative score thus ranged from 0 to 7. A total score exceeding 5 or more was classified as indicating good knowledge, whereas a score of 4 or less represented poor knowledge. The cutoff was determined through consensus among two senior faculty members—one from the Pediatrics discipline and one from Community Medicine—based on expert judgment and contextual relevance. (C) The attitude assessment component consisted of eight items with Agree/Disagree responses; and (D) The practice assessment component included nine Yes/No questions focusing on home-based management and preventive practices. The categorization of diarrhea management practices into good, fair, or poor was guided by WHO/UNICEF-recommended strategies (17) (Supplementary Table 2).

Assessment approaches include the use of ORS, increased fluid intake, continued feeding, a combination of all treatment modalities (ORS, continued feeding, and extra fluids), and any combination of modalities during diarrheal episodes.

### Data analysis

Data was entered in Excel and analyzed using SPSS version 23. Descriptive statistics (frequencies, percentages, means, and standard deviations) summarized study variables. Logistic regression assessed associations between mothers’ knowledge of diarrhea management and socio-demographic factors.

## Results

Present study explored mothers’ knowledge, beliefs, and practices regarding diarrhea prevention and management in children under-five in Pune, Western Maharashtra, India. 122 mothers, averaging 29.73 (± 2.91) years (SD), with children averaging 37.9 (±12.56) months (SD) were involved. They lived in households with an average of 4 members and 2 children under-five years. The majority of mothers (72.1%) were employed, 47.5% had a graduate degree, and 48.3% had their family income between ₹31,000 and ₹50,000 each month.

### Mothers’ knowledge on prevention of diarrhea in children

This study assessed mothers’ understanding of treating and preventing diarrhea in children under-five by asking them multiple-choice questions regarding the causes, symptoms, and risks of the condition. Majority of the mothers (67.2%) accurately defined diarrhea as three or more watery stools per day, but 13.1% of them were unaware of the same. Nearly all (99.18%) opted contaminated water as the primary cause of diarrhea followed by contaminated food (90.1%) and infection (61.47%). Contaminated water (96.72%), lack of vaccination (34.6%), unhygienic practices (38.0%) were mentioned as main reasons for transmission of diarrhea. 97.5% of the mothers identified dry lips and tongue while 87.7% mentioned inadequate eating or drinking as symptoms of dehydration.

When mothers’ knowledge was correlated to their sociodemographic characteristics, it was found that mother participants who were educated above schooling level (*p* < 0.001), were employed (*p* < 0.01), or had higher (> 30,000/− per month) family income (*p* = 0.02) knew significantly better about diarrhea and how to treat it (Table 1).

**Table 1:** Correlation of mothers’ knowledge about diarrhea to their sociodemographic characteristics.

Variables	Poor (*n* = 32, 26.2%)	Good (*n* = 90, 73.8%)	Total	Chi-square	*p* value
Mother age (years)					
20–24	3 (75)	1 (25)	4 (3.28)	6.86	0.07
25–29	9 (18.8)	39 (60.12)	48 (53.28)		
30–34	18 (27.7)	47 (72.3)	65 (53.28)		
35+	2 (40)	3 (60)	5 (4.10)		
Mother’s education					
Graduation and above	17 (19.3)	71 (80.6)	88 (72.13)	13.7	0.001*
12th grade	5 (27.8)	13 (72.2)	18 (14.75)		
10th grade or below	10 (62.5)	6 (37.5)	16 (13.11)		
Occupation of mother					
Employed	9 (15.5)	49 (84.5)	58 (47.54)	6.55	0.01*
Unemployed	23 (35.9)	41 (64)	64 (52.46)		
Family monthly income (Indian rupee)					
<5,000	2 (6.9)	27 (93.1)	29 (23.77)	11.14	0.02*
5,000–10,000	2 (66.7)	1 (33.3)	3 (2.46)		
11,000–20,000	3 (50)	3 (50)	6 (4.92)		
21,000–30,000	9 (36)	16 (64)	25 (20.49)		
31,000–50,000	16 (27.1)	43 (72.9)	59 (48.36)		
Number of family members					
≤ 4	13 (21.0)	49 (79.1)	62 (50.82)	1.8	0.17
> 4	19 (31.7)	41 (68.3)	60 (54.9)		

* < 0.05 (Chi square test).

### Mothers’ attitude toward management and prevention of diarrhea

95% of participant mothers agreed that diarrhea is a serious health issue and 86% thought that it is preventable and manageable at home. The benefits of ORS in diarrhea were widely accepted among all mothers. This included its crucial role in preventing salt and water depletion, as stated by 106 (86.9%) mothers, and 112 (92%) knew that ORS could be homemade.

**Figure 1:**
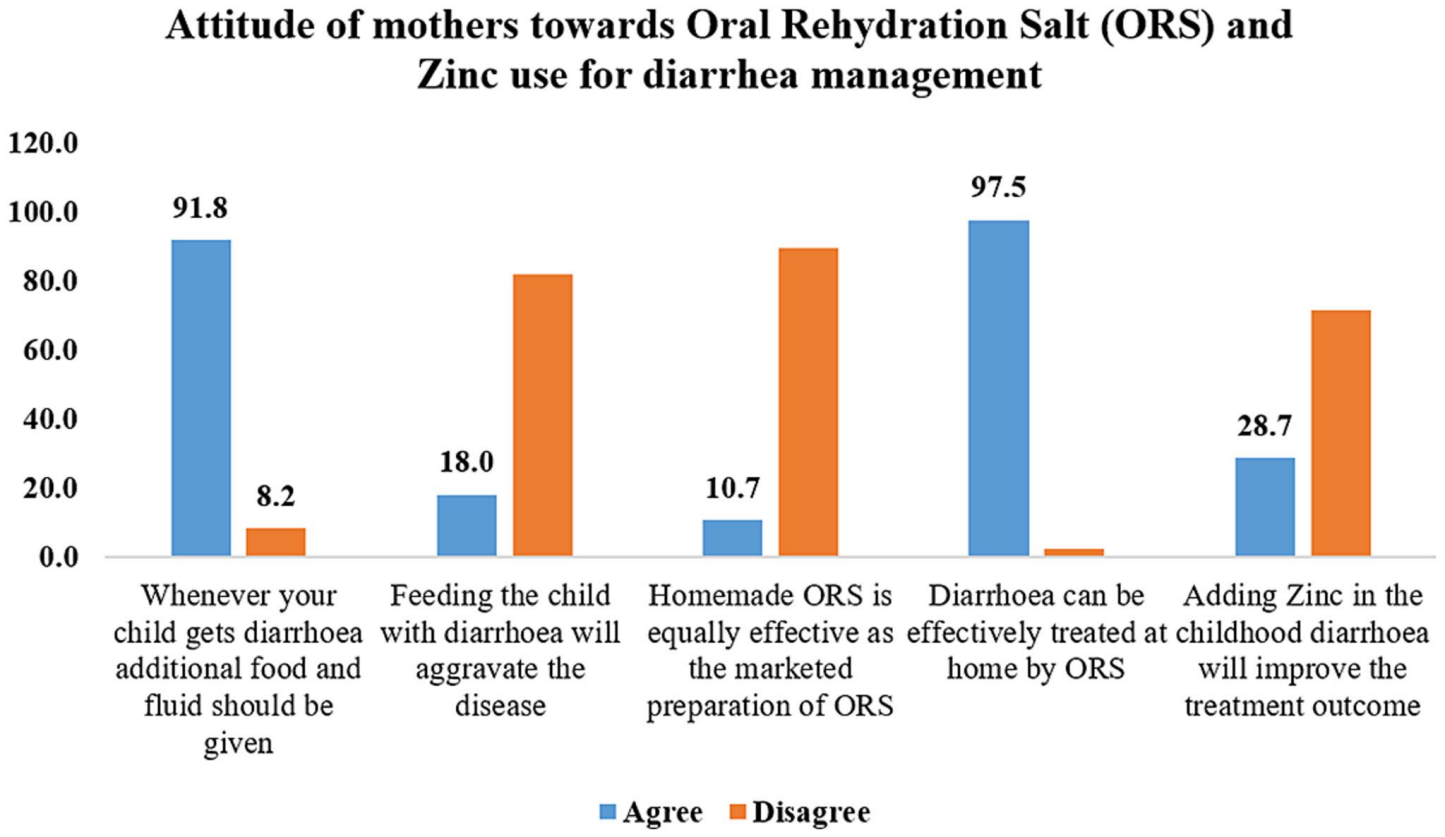
Mothers’ attitudes toward oral rehydration solution and zinc use in diarrhea.

Although all mothers agreed on the beneficial role of Oral Rehydration Solution (ORS) in diarrhea treatment, their perspectives on other therapies varied. 81.96% of the participants believed that continuing to feed a child with diarrhea would prevent the illness from getting worst. Moreover, 91.8% felt it was crucial to provide more food and water to a child experiencing diarrhea (Figure 1). In contrast 10.7% participants considered homemade oral rehydration solutions to be as effective as commercially available ORS and only 28.7% were of the opinion that zinc supplementation would enhance treatment outcomes.

In the current maternal survey, which permitted multiple responses, 85.24% mothers reported continuing to feed their child during a diarrheal episode. While 2.13% mothers offered a typical normal family diet, nearly 18% provided alternatives such as rice water, coconut water, soup, or sago to their child during diarrheal episode. Knowledge of correct ORS preparation was good, with 96% respondents demonstrating this understanding, and 97.54% mothers knowing the appropriate duration for storing prepared ORS. Regarding frequency of ORS administration for children under-five with diarrhea, 68.85% correctly stated that they administered ORS after each loose stool. Unfortunately, awareness of zinc’s benefits in diarrhea treatment and its appropriate duration was limited, with only 28.68% possessing this knowledge (Table 2).

**Table 2:** Home-based practices among mothers for children with diarrhea.

Variables	Category	Frequency	Percentage (%)
What should be given when your child has diarrhea? (*N* = 609) (Multiple responses possible)	Normal diet	13	2.13
	Rice water	108	17.8
	Sago	109	17.9
	Boiled potato	103	16.9
	Soup	104	17.1
	Coconut water	106	17.4
	Fruit juice	66	10.83
What water do you use to mix ORS solution?	Previously boiled and cooled water	117	96
	Any available water	0	0
	Mineral water	5	4.09
If your child started diarrhea what you will do?	Stop feeding	18	14.75
	Continues feeding	104	85.24
Frequency of giving ORS	2–3 times a day	13	10.65
	4–5 times a day	25	20.49
	After every loose stool	84	68.85
Amount of ORS during a diarrheal episode	As much as the child can drink	111	90.98
	A Cup approx. 100 mL	9	7.37
	No idea	2	1.63
Prepared ORS duration of stay	24 h (1 day)	119	97.54
	More than 24 h	3	2.45
Frequency of giving Zinc to your child in diarrhea	Till diarrhea stops	7	5.73
	14 days even after diarrhea stops	29	23.77
	No idea	86	70.49

When the practices of participant mothers for management of diarrhea were correlated to their sociodemographic variables, no significant association was found between their practices with education, employment or monthly family income (Table 3).

**Table 3:** Correlation of Mothers’ practice score about diarrhea to their sociodemographic characteristics.

Variables	Practice score			Chi square	*p* value
	Fair Practice	Poor Practice	Total (*n* = 122)		
Mother age (years)					
20–24	4	0	4	1.26	0.73
25–29	43	5	48		
30–34	57	8	65		
35+	5	0	5		
Child’s age (months)					
≤12	3	1	4	2.84	0.58
13–24	16	1	17		
25–36	18	4	22		
37–48	52	5	57		
>48	20	2	22		
Number of family members					
≤ 4	55	7	62	0.05	0.81
> 4	54	6	60		
Educational status of mother					
Graduation and above	80	8	88	3.08	0.21
Higher secondary	14	4	18		
Secondary schooling	15	1	16		
Occupation of mother					
Employed	55	3	58	3.49	0.06
Unemployed	54	10	64		
Family monthly income (Indian rupee)					
<5,000	29	0	29	5.31	0.25
5,000–10,000	3	0	3		
11,000–20,000	5	1	6		
21,000–30,000	21	4	25		
31,000–50,000	51	8	59		

According to WHO/UNICEF recommendations for treatment of diarrhea, assessment approaches including use of ORS, increased fluid intake, continued feeding, in combination, can be categorized into three levels: good, fair, and poor practice. 38% of mothers had fair practices, whereas nearly 90% of mothers knew the advantages of extra fluids and ORS and had good practices for managing diarrhea. Out of 10% mothers with poor management practices, 3% did not administer ORS, extra fluids, or ongoing feedings to their child during diarrheal episode.

**Figure 2:**
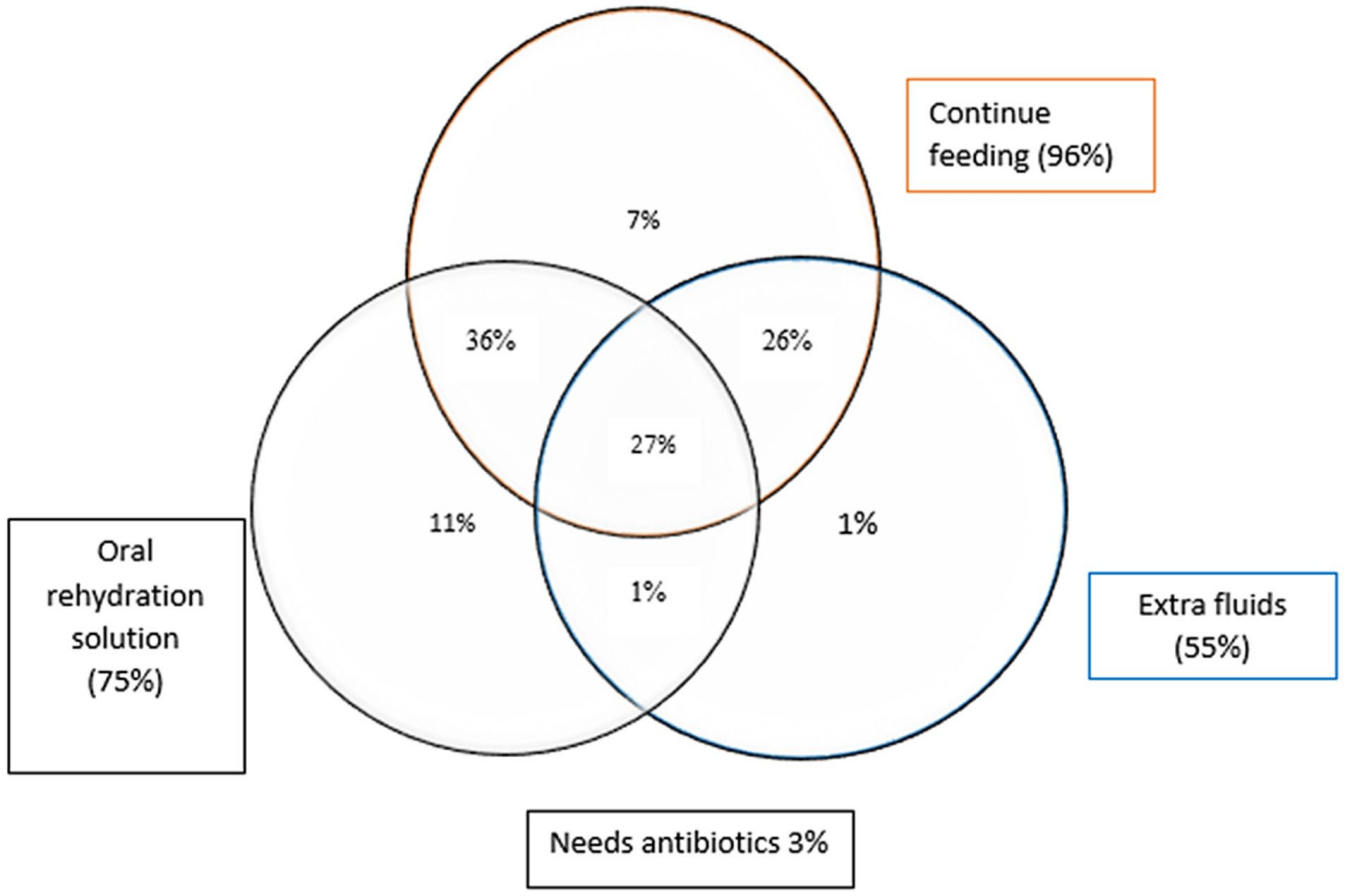
The management approach for childhood diarrhea: percentage of children receiving Oral Rehydration Solution, continue feeding, and/or are given extra fluids.

Additionally, the Venn diagram (Figure 2) represents the diverse treatment approaches our participant mothers used, underscoring the complex nature of decision-making in diarrhea management. All the treatment modalities are depicted in the diagram: 36, 26, and 1% children received ORS and continued feeding, additional fluids and continuous feeding, and ORS and further fluids, respectively. Notably, 3% of mothers relied solely on antibiotic treatment, opting out of all three standard approaches. Conversely, 27% of children benefited from a comprehensive strategy, receiving ORS, additional fluids, and continued feeding.

**Figure 3:**
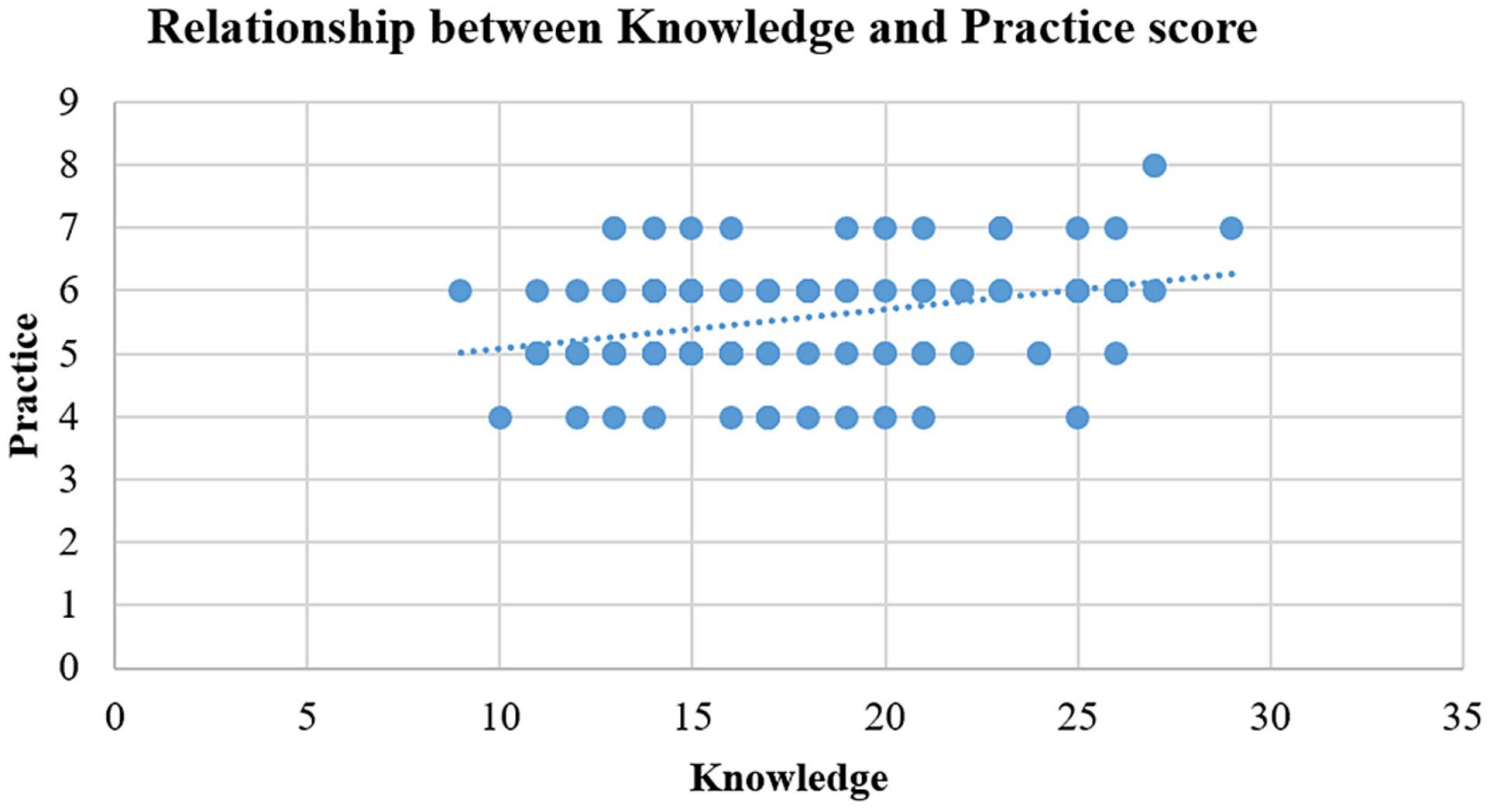
Correlation of mothers’ knowledge score with their practice score for diarrhea management in under-five children.

The scatter plot (Figure 3) shows a slight positive association between mothers’ practices and knowledge regarding management of diarrhea in their under-five children. According to the coefficient of determination (*r* = 0.34, *r*^2^ = 0.117), mothers’ knowledge level explains around 11.7% of the variation in their practices, which is a comparatively small percentage of the total variation in mothers’ practices.

## Discussion

Diarrhea remains a leading cause of illness and death among children under the age of five worldwide. However, a significant proportion of these deaths and illnesses are preventable. Understanding the signs and symptoms, transmission, and prevention of diarrhea, along with adopting healthy practices like immunization, exclusive breastfeeding, using ORS, and ensuring good sanitation, is vital to reducing both morbidity and mortality. Timely and proper management at both the household level and within healthcare settings plays a crucial role in addressing this issue. To assess how well mothers of under-five children understand and manage diarrhea, the present study was conducted to evaluate their knowledge, attitudes, and practices regarding its management.

A total of 122 mothers with children under the age of five were interviewed and included in the study. This study involved mothers with an average age of 29.73 years, and majority (72.1%) held a graduate degree or higher, suggesting a youthful cohort and strong health literacy potential. Regarding employment, nearly 50% of the mothers were employed, and a similar proportion (48.3%) reported a monthly household income between ₹31,000 and ₹50,000. These economic and employment factors can affect the mothers’ access to healthcare, ability to prioritize health, and the financial resources available for medical expenses or health-related activities.

The present study showed that mothers had a good understanding of diarrheal diseases. 62.7% of mothers correctly defined diarrhea according to the description provided by the WHO (18). When compared to other studies, the proportion of mothers with a correct understanding of diarrhea varies significantly both within India and globally. For example, a study conducted in the eastern part of Delhi by Chaudhary P et al. reported a much higher proportion of 96% mothers being able to define diarrhea correctly (19). In contrast, studies from other regions in India mentioned that the concept of diarrhea was clearly understood by only 24% participants in a study from Mangalore and 94% in the study from rural part of Karnataka (20, 21). International studies also show differing levels of awareness, with 35% of mothers in Sudan, 65% in the Philippines, and 79% in Nepal correctly defining diarrhoea (22–24).

In present study, nearly all mothers (99.18%) stated that contaminated water and food were the most common causes and mode of spread of diarrhea in children under the age of 5 years. Also, unhygienic practices (38.3%) and non-immunization (34.6%) were most commonly stated predisposing factors for diarrhea by mothers. Significant finding from a study by Padhy et al. was the knowledge gaps among mothers regarding common childhood illnesses like diarrhea where only 52% of mothers understood its causes, and 58% were aware of risk factors. These findings mirror those from other study by Hackett et al. (13) where only 47% of mothers were unaware of the causes of diarrhea. In current study, most of the mothers knew the danger signs and symptoms of dehydration related to diarrhea as dry lips and tongue (97.5%) and eating or drinking poorly (87.7%). Whereas study carried out in Karnataka, India reported that mothers had limited knowledge regarding danger signs of diarrhea as only 39.71% of the study participants identified at least one danger sign of diarrhea (25). Similarly, the study from Delhi also revealed that 36% of mothers had no understanding of it (11), the findings of both studies align with reports from other research conducted in India and across Asia (22, 24). Most of them were ignorant about the danger signs of diarrhea and reinforce that, highlighting the need of health education of mothers in this regard may be of helpful (25).

Mothers participating in current study recognized the benefits of ORS for diarrhea, and 92% mentioned to prepare ORS powder correctly with the recommended amount of water. These results are similar to an Ethiopian study in which 85.4% of carers properly prepared ORS and 73.2% of them utilized it as soon as diarrhea started. Similarly, a study conducted in Bangladesh found that 88.9% of carers used ORS, indicating a strong link between ORS use and maternal education. Additionally, Chaudhary P et al. found that a higher percentage of mothers had sufficient understanding about ORS preparation and gave extra fluids (19). Similar findings, which show a typically high level of understanding regarding the proper use of ORS, have been reported in several Indian studies as well as international research from Iran, Cambodia, and Nepal (26–28). The importance of zinc supplementation with ORS for improving treatment outcome, was understood only by 28.7% participants in the current study (Figure 1). Similar observations are reported by previous studies conducted in Maharashtra, India by Datta V et al. and Mallick AKR et al. where the participants’ understanding of the use of home-based rehydration solutions, the beneficial effects of zinc supplementation and their ability to recognize signs of dehydration were inadequate (29, 30).

Interestingly, a review suggested that the underuse of ORS may be linked to healthcare practitioners’ tendency to prescribe antibiotics rather than prioritize ORS as the primary treatment (31). This is particularly relevant when rotavirus diarrhea is responsible for 40% of childhood hospitalizations annually and it has been estimated that approximately 23% of diarrheal deaths in India occur due to rotavirus infection (32). A positive observation from the present study was that only 3% of mothers believed that antibiotic treatment was the only way to manage their child’s diarrhea (Figure 2). This differed from a study conducted in a rural area of Egypt, which reported that antibiotics were given to about two-thirds of children ill with diarrhea, and about half of the children received antidiarrheal drugs (33). This finding is similar with that of the study conducted in Kenya, which revealed that more than 50% of the mothers frequently used antibiotics to manage diarrhea episodes (34). The reasons cited for the high use of antibiotics and antidiarrheal drugs included caregivers’ belief that they were accessible, efficacious, and widely available, typically without a prescription (35). In current survey, most of participants (81.96%) agreed that feeding a child during diarrhea helps prevent its worsening, and 91.8% believed additional food and fluids should be provided. These findings are considerably better than those reported studies by other Indian authors (30, 36, 37). According to WHO/UNICEF guidelines, findings in our study show that nearly 90% of mothers followed good practices, especially in using ORS and maintaining fluid intake. While feeding continuity and correct ORS preparation were well adopted, zinc supplementation remained underutilized. Addressing this gap requires coordinated efforts to enhance caregiver awareness, improve accessibility to essential treatments, and reinforce health systems. In contrast results from Barakat A et al. revealed that only 11% of children received all three recommended treatments, while 7% received none; overall, 36% of mothers managed diarrhea effectively, with 56% failing to do so (38).

Mothers’ knowledge when correlated with sociodemographic variables showed that mothers with higher education, employed and with more family income, had a better level of knowledge on understanding of diarrhea and its management (Table 1). Mothers with higher education are more likely to be informed about health issues and adopt health-promoting behaviors. Similarly, a study by Joseph et al. in Karad, Maharashtra, found that mothers initially had inadequate knowledge regarding the prevention and home management of diarrhea. However, after receiving health education, their knowledge improved significantly, highlighting the effectiveness of the educational intervention (39).

There was no significant association between diarrhea management practices among the participant mothers with their sociodemographic factors like education, employment, or income (Table 3). This indicates a need for targeted education to address knowledge gaps and increase confidence in proper diarrhea management, which could improve health outcomes for children at risk of dehydration and its complications. In a study carried out by Rokkappanavar KK et al., the authors discovered a significant link between the education level of mothers and their proper practices in managing diarrhea, hence, they emphasized the importance of health education (36). Based on above observations, interventions should be universally targeted at all mothers, regardless of socioeconomic status which could help reduce morbidity and mortality in children under 5 years.

In current study, a weak positive correlation (*r* = 0.34) was found between mothers’ knowledge and practices in managing diarrhea (Figure 3), suggesting that improved knowledge generally leads to better practices. However, this correlation is not strong, indicating that knowledge alone does not fully explain maternal behaviors. Several factors, including cultural beliefs, healthcare accessibility, and personal attitudes, also influence how mothers apply their knowledge in practice (40–43). Additionally, individual perceptions of diarrhea severity and treatment effectiveness shape decision-making and practice consistency.

## Data Availability

The original contributions presented in the study are included in the article/Supplementary material, further inquiries can be directed to the corresponding author.
