# Supplementary material for: Maternal practices and awareness of diarrhea management in children under-five: evidence from Pune, India
Source: Front Public Health. 2025 Dec 8;13:1673617. doi: 10.3389/fpubh.2025.1673617 (PMC12719074; doi:10.3389/fpubh.2025.1673617)
Supplement: Supplementary file 1 [file Table_1.docx]

**Supplementary Table 1- KAP of mothers in prevention and home-based management of diarrhea in under five children**

PART I. Demographic Status

| ***S.NO*** | | ***QUESTIONS*** | | ***RESPONSES*** |
| --- | --- | --- | --- | --- |
|  | Age of the mother (Years) | | 15–24  25–34  35–44  > 45 | |
|  | Educational status of mother | | Illiterate  primary schooling  secondary schooling  Graduation and above | |
|  | Occupation of mother | | Housewife  Employed | |
|  | Total No.of family members | | 1-2  3-4  ≥5 | |
|  | Total No.of children in the family with age ≤ five years | | 1  2  3  ≥ 4 | |
|  | How much is your family monthly income?(In Rupees) | | 5000 -10000  11000-20000  21000-30000  31000-50000  ≥50000 | |
|  | Status of your child for | | Measles vaccination  MMR vaccination  Rotavirus vaccination  Vitamin A doses | |

**PART II: KNOWLEDGE OF MOTHER ABOUT DIARRHEA PREVENTION AND HOME BASED MANAGEMENT IN THEIR UNDER FIVE CHILDREN**

**Note: Please tick the appropriate answer. More than one answer will be accepted wherever applicable**

| ***S.NO*** | ***QUESTIONS*** | ***RESPONSES*** |
| --- | --- | --- |
| 1 | What do you understand by diarrhea? | Frequent passing of watery stool  (3 or more times)  Blood in stools  Mucus in stools  No idea  Others, please specify………….. |
| 2 | What do you think are the causes of diarrhea? | Infection  Food Poisoning  Teething / Crawling  Worm Infestation  Evil spirits  Contaminated water  Poor digestion & absorption of food  drug side effects  No Idea  Others; please specify………… |
| 3 | Modes of the spread of infection | Contaminated water  Spoiled or contaminated food  Flies  Bottle feeds  Unclean fingers and hand  No Idea  Others; please specify………… |
| 4 | Predisposing Factors | Undernourished/underweight child  Unhygienic practices  Measles infection  Vitamin deficiency  Non-immunization  Don’t know |
| 5 | What are some of the common signs associated with diarrhea? | Frequent passing of watery stools  May have vomiting with diarrhoea  May have fever with diarrhoea  May have Blood/mucus in the stool |
| 6 | What are the Signs of Dehydration? | Marked thirst for water  Eating or drinking poorly  Sunken eyes  Dry Lips and tongue  Reduced urine output  Reduced skin turgor  Irritability  Lethargic  Don’t know |
| 7 | What are the complications of unmanaged diarrhoea (Needs doctor’s consultation)? | Severe dehydration  Severe irritability  Unable to move  Unconscious  Convulsions  Don’t know  Others, please specify …………… |

PART III. ASSESSMENT OF Part III: ATTITUDE OF MOTHERS TOWARD PREVENTION AND MANAGEMENT OF DIARRHEA IN THEIR UNDER FIVE CHILDREN

Do you think-

| ***S.NO*** | ***QUESTIONS*** | ***RESPONSES*** |
| --- | --- | --- |
| 1 | Diarrhea is serious health problem | AGREE DISAGREE |
| 2 | Diarrhea is preventable disease and It is manageable at home | AGREE DISAGREE |
| 3 | Giving child ORS during diarrhoea is benefit | AGREE DISAGREE |
| 4 | Mothers can prepare oral rehydration at home | AGREE DISAGREE |
| 5 | Giving Oral rehydration fluids at home can treat diarrhea | AGREE DISAGREE |
| 6 | Oral rehydration fluids replenishes the salt and water lost in diarrhea | AGREE DISAGREE |
| 7 | Giving child ORS during diarrhoea reduces severity and duration of diarrheal disease | AGREE DISAGREE |
| 8 | No other treatment other than ORT needed for most of the diarrhoea | AGREE DISAGREE |
| 9 | Giving Zinc is helpful in diarrhoea | AGREE DISAGREE |

**PART IV. ASSESSMENT OF PRACTICE OF MOTHERS IN PREVENTION AND MANAGEMENT OF DIARRHEA IN THEIR CHILDREN**

**Note: Please tick the appropriate answer. More than one answer will be accepted wherever applicable**

| 1 | How do you prepare oral rehydration therapy at home? | Use homemade ORS (with salt and water)  Use sachet of ORS (powdered ORS)  Use Tetra pack of ORS |
| --- | --- | --- |
| 2 | What sort of water you use while preparing ORS | Tap water  Filtered water  Mineral water  Boiled and cooled water |
| 3 | How you prepare marketed ORS at  Home? | Small sachet - Mix ORS in 1 glass of water  Big Sachet - Mix ORS in 1 liter of water  Mix ORS in water without any measurement  Follow the instructions on sachet |
| 4 | When do you give ORS to your child in diarrhoea? | To make out (balances) of the lost fluid  After every watery stool  Once a day  2-3 Times a day  Wherever the child wants to drink  No idea |
| 5 | Once prepared, how long the ORS can be used? | 24 hrs. (1 day)  More than 24 hrs. (≥ 1 day)  Others please specify ………… |
| 6 | When do you give Zinc to your child in diarrhoea? | Till diarrhoea stops  14 days even after diarrhoea stops  No idea  Others please specify ………… |
| 7 | What other treatments can be offered at home to a child in diarrhoea? | If child is on breast feed  Stop breast feeding  Continue breast feeding  If child is eating  Stop giving food  Continue giving food |
| 8 | What treatments can be offered to a child in diarrhoea? | Must receive increased fluids,ORS, zinc, and regular feeding  Requires ORS, but should receive less food in order to reduce the diarrhoea  Should immediately receive antibiotics to stop the diarrhoea |
| 9 | What oral feeds can be given if child has diarrhoea? | Rice water  Sweetened commercial fruit drink  Soup  Sago  Rice kanji  Boiled potato  Lemon drink  Coconut water  A soft drink  Others Please specify ………………… |

**Supplementary Table 2-** Classification of diarrhoea management practices based on WHO/UNICEF recommended interventions

| **Classification**  **(Practice)** | **Child given ORS** | **Child given**  **increased fluids** | **Child continued**  **feeding** |
| --- | --- | --- | --- |
| Good | Yes | Yes | Yes |
| Good | Yes | Yes | No |
| Good | Yes | No | Yes |
| Fair | Yes | No | No |
| Fair | No | Yes | Yes |
| Fair | No | Yes | No |
| Poor | No | No | Yes |
| Poor | No | No | No |
